# Pulmonary Alveolar Proteinosis in Association with Congenital Dyserythropoietic Anemia: A Case Report

**DOI:** 10.1155/2012/624740

**Published:** 2012-06-28

**Authors:** Marcus A. Carden, Ashish Barman, Gita Massey

**Affiliations:** ^1^Departments of Internal Medicine and Pediatrics, Virginia Commonwealth University, 1001 East Marshall Street, Richmond, VA 23298-0646, USA; ^2^Division of Anatomic Pathology, Department of Pathology, Virginia Commonwealth University, Richmond, VA 23298-0646, USA; ^3^Division of Pediatric Hematology and Oncology, Department of Pediatrics, Children's Hospital of Richmond, Virginia Commonwealth University, Richmond, VA 23298-0646, USA

## Abstract

A two-year-old girl with congenital dyserythropoietic anemia (CDA) acutely developed fever, tachypnea, and increased oxygen requirement. Chest X-ray revealed bilateral interstitial infiltrates and mild cardiomegaly. Blood cultures grew no infectious agents, while pulmonary specimens grew cytomegalovirus (CMV). Treatment with intravenous ganciclovir was initiated but without response. Final cytologic preparations of bronchoalveolar lavage (BAL) fluid revealed eosinophilic amorphous material consistent with pulmonary alveolar proteinosis (PAP). CDA and PAP are extremely rare disorders in pediatrics. PAP should be considered in patients with hematological disorders who present with acute interstitial pneumonia, after infectious causes are ruled out.

## 1. Introduction

Congenital dyserythropoietic anemia type II (CDA II), also known as HEMPAS (hereditary erythroblastic multinuclearity with positive acid serum test), is a rare hematological autosomal recessive disorder whereby individuals have bone marrow biopsy findings indicating inappropriate red blood cell lineage progression [1]. Pulmonary alveolar proteinosis (PAP) is also a rare disorder in which lipoproteinaceous material congregates within alveolar spaces, leading to a host with impaired pulmonary immunity and susceptibility to opportunistic infection [2]. Although historically reported in patients with various hematological disorders [3–8], to our knowledge, this is the first case report of PAP associated with CDA.

## 2. Case Report

A 23-month-old girl presented to our facility with one week of worsening fevers, productive cough, and increased work of breathing. Over the previous 3 months, she was treated for presumed upper respiratory tract infections with inhaled beta-agonists, inhaled steroids, oral steroids, and several different oral antibiotics. Relevant medical history was significant for prematurity and severe anemia necessitating intrauterine blood transfusions, CDA diagnosed at 20 months by bone marrow biopsy and aspiration that was morphologically consistent with type II, and surgical placement of a Port-a-Cath for chronic blood transfusions.

Initial vital signs were temperature 37.9 degrees Celsius, heart rate 156 bpm, respiratory rate 50/min with oxygen saturation 83% on room air and 98% on 1 LPM oxygen by nasal cannula, and blood pressure 115/61 mm Hg. She appeared in mild distress with increased work of breathing. She had moderate frontal bossing with open anterior fontanelle. Heart examination was significant for a II/IV systolic ejection murmur. Lung exam revealed diffuse rales bilaterally but no wheezing. Abdomen was not distended. The liver was palpated 4 cm below the costal margin, and the spleen palpated to the level of the umbilicus. She had no clubbing, cyanosis, or peripheral edema.

**Figure 1:**
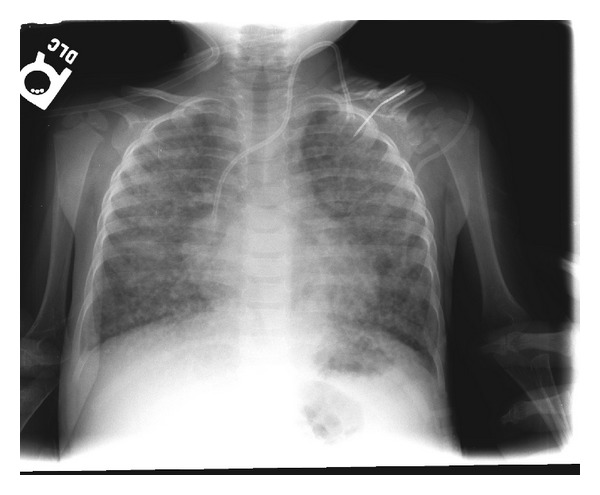
PA radiograph on admission: well-inflated lungs with diffuse bilateral coarse reticular opacities and left IJ port.

Chest radiograph showed diffuse ground glass alveolar infiltrates which were chronic in nature when compared to a chest X-ray from one year prior (Figure 1). Blood, urine, and respiratory cultures were sent, and the patient was started empirically on oral azithromycin and parenteral ceftriaxone.

On day 3, a transthoracic echocardiogram revealed a patent foramen ovale but no cardiomyopathic process. An abdominal ultrasound confirmed hepatosplenomegaly. Repeat chest X-ray showed no changes, and chest CT was offered but the parents refused.

**Figure 2:**
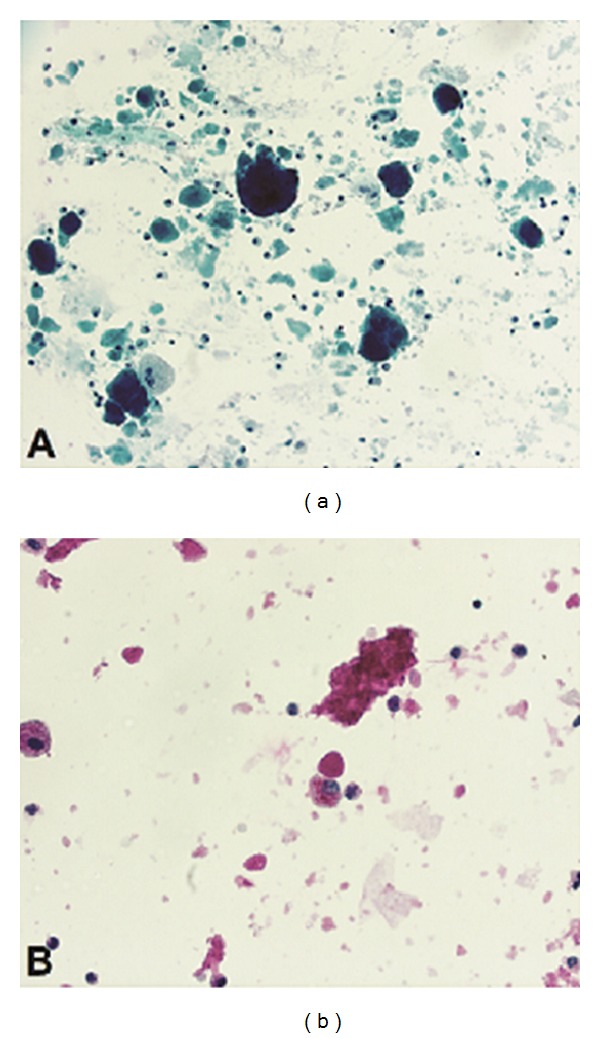
Evidence of pulmonary alveolar proteinosis: papanicolaou stain of bronchoalveolar lavage (BAL) fluid at medium power (20x) view showing large, amorphous, acellular aggregates with a pulmonary macrophage adjacent to an aggregate in a background of chronic inflammatory cells (a). Periodic acid-Schiff (PAS) histochemical stain of BAL fluid at high power (40x) highlights both the large extracellular amorphous aggregates as well as material within pulmonary macrophages (b).

Despite 4 days of antibiotics, the patient continued to spike intermittent fevers and was still on oxygen treatment, and there still were no changes on chest radiograph. Bronchoscopy was performed and was anatomically normal. The bronchoalveolar lavage (BAL) fluid was cloudy in appearance and revealed no evidence of aspiration or malignant cells. Grocott's methenamine silver (GMS) stain was negative for *Pneumocystis jirovecii*. Ziehl-Neelsen stain was negative for acid-fast bacilli (AFB). Fungal, bacterial, AFB, RSV, and influenza cultures of BAL fluid revealed no growth. Differential of the WBCs indicated 68% macrophages, 12% PMNs, and 10% lymphocytes. Light microscopic examination of BAL fluid using Periodic acid-Schiff (PAS) stain was positive for abundant amorphous material within the pulmonary macrophages and also in larger extracellular aggregates suggesting pulmonary alveolar proteinosis (Figure 2). The lipid-laden macrophage (LLM) index was 120 [9].

On day 5, the patient's work of breathing improved, lung sounds improved, and the supplemental oxygen was decreased to 0.5 LPM by nasal cannula. Antibacterials were discontinued. On day 7, BAL fluid was positive for cytomegalovirus (CMV) by indirect fluorescent antibody testing and serum CMV IgG and IgM antibodies were detected by immunoassay, while CMV DNA of the blood remained negative. Ophthalmologic examination revealed no signs of CMV retinitis. The patient was started on parenteral ganciclovir. On day 9, the patient was weaned off supplemental oxygen to room air. She continued IV ganciclovir and was discharged from the hospital.

After completion of two weeks of ganciclovir, the patient presented to her hematologist's office for scheduled followup and blood transfusion. Chest X-ray was repeated and showed minimal interval improvement in the diffuse coarse reticular opacities noted on X-ray from her most recent hospitalization. Again, the parents refused CT scan. The patient was subsequently lost to followup. Several months later she was admitted to a hospital in another state in respiratory failure. Per report, she required intubation and BAL was performed, which confirmed PAP.

## 3. Discussion

Our patient presented with concerns of imminent respiratory failure and diffuse interstitial infiltrates on chest X-ray. With the source unidentified by noninvasive means, and with continuing illness despite medical management, BAL was performed for diagnostic purposes. Initial findings suggested that CMV was the culprit in our patient with apparent bilateral pneumonia. However, lavage fluid also had findings consistent with PAP. Despite adequate treatment with ganciclovir, the patient continued to have concerning respiratory findings and a chest X-ray that remained essentially the same. Physicians should consider BAL as both a diagnostic and therapeutic tool early in the course of a patient with hematological pathology presenting with diffuse pulmonary infiltrates with unknown etiology.

The congenital dyserythropoietic anemias are very rare disorders that can be divided into three groups: CDA I–III. The morphological aberrancies of CDA II, which include binucleated and multinucleated RBC precursor cells with membrane irregularities as seen on our patient's diagnostic bone marrow sample, are thought to be secondary to hypoglycosylation of erythrocyte membrane proteins [10–12]. Complications that have been reported include splenomegaly, gallstones, jaundice, and pulmonary failure [13].

PAP is a rare disorder involving deposition of lipoproteinaceous material in alveoli and results in a deficit of surfactant clearance by pulmonary macrophages [3]. There are 3 types: acquired, secondary, and congenital. Secondary PAP is associated with hematological malignancies and is well-established in the literature [14]. Superimposed infection is known to complicate the course of secondary PAP, and reports of CMV and PAP have been reported [15].

To our knowledge, however, there are no published accounts of PAP associated with CDA and the incidence of these two rare disorders occurring simultaneously is remarkable and elucidating a possible mechanism is intriguing. While the acquired form of PAP is associated with an autoimmune process directed at the granulocyte-macrophage colony-stimulating factor (GM-CSF), resulting in suppression of proper alveolar function, the congenital type is associated with a rare mutation in the GM-CSF receptor [3, 16, 17]. Along those same lines, secondary PAP in association with hematological disorders such as CDA II could result from defective GM-CSF receptors on pulmonary macrophages and/or in a disrupted signal transduction pathway after GM-CSF and receptor interaction, which has been proposed elsewhere [8]. It is possible, as well, that our patient had a functional impairment of alveolar macrophages resulting from the environmental disruption of normal robust monocyte production caused by pathogenic erythrocyte production inherent to CDA II. Glycosylation defects of the monocytes themselves are less likely as it has been shown such errors are limited to erythroblasts [12].

The success of whole-lung lavage to relieve symptoms in secondary PAP has been documented thoroughly [2]. The sudden improvement of our patient after BAL supports this, and the lack of improvement after adequate antiviral therapy argues against CMV as the primary culprit. Experts feel CMV, and other infectious agents isolated in patients with secondary PAP and hematopoietic aberrancy are likely opportunistic infections superimposed on a preexistent PAP and compromised immune system [18]. The diagnosis of PAP alone, or in combination with superimposed CMV infection, should be entertained in any pediatric patient with a known hematological condition and new respiratory changes with a chest X-ray showing diffuse lung disease.
